# Italian neonatal birthweight charts derived from INeS not separated by birth order

**DOI:** 10.1186/s13052-024-01660-7

**Published:** 2024-04-29

**Authors:** Elena Spada, Chiara Peila, Alessandra Coscia

**Affiliations:** Department of Public Health and Pediatrics, Neonatal Unit, University of Turin, Turin, Italy

## Abstract

**Background:**

Identifying high-risk neonates with abnormal fetal growth is crucial for health risk prediction and early intervention. Small for gestational age (SGA) and large for gestational age (LGA) classifications highlight neonates having a higher risk for postnatal diseases. Accurate diagnosis depends on precise anthropometric measurements and appropriate reference data. In 2010, specific neonatal charts for Italian singletons (INeS charts) were published, tracing separately for first- and later-born neonates due to a 3% birth weight difference. We present INeS charts for birth weight non-separated by first- and later-born babies useful when information on parity is unavailable or unreliable, or for better comparisons with other neonatal charts that are not separated by birth-order.

**Methods:**

INeS charts were traced using a parametric function. Starting with the parameters estimates published in a different paper, INeS charts not separated by birth order were traced for the gestational age range of 23 to 42 weeks. In a second step the charts were parametrized as Cole and Green Lambda Mu and Sigma (LMS) model, allowing computation of standard deviation scores.

**Results:**

The centiles of non-separated INeS charts follow between first- and later-born charts. Distances varied due to changing first-born proportions with gestational age, Max differences of about 100g with later born and 70g with first-born were observed at term. S and L functions have a similar shape for boys and girls. S function shows a pick at about 29 weeks, L function has positive values in all the range of gestational age with a pick at 39 weeks.

**Conclusions:**

The study presents non-separated Birth Weight INeS charts, bridging the gap when parity information is unavailable. Differences with separated charts were generally small, making them reliable for neonatal health assessment. Insights from L and S parameters contribute to standardized birth weight and adjust it by sex and Gestational Age, useful for defining SGA or LGA neonates. The paper enhances neonatal care tools, showcasing INeS chart flexibility in different clinical scenarios and supporting neonatology research.

**Supplementary Information:**

The online version contains supplementary material available at 10.1186/s13052-024-01660-7.

## Background

The effective identification of high-risk neonates with abnormal fetal growth plays an important role in health risk prediction, Prognosis assessment and early intervention [1].

Small for Gestational Age (SGA) and Large for Gestational Age (LGA) describe neonates born with a birth weight below or above defined cut-offs for gestational age (generally the 10^th^ and the 90^th^ centile respectively). This classification identified two categories of neonates having a higher risk for postnatal multiple diseases, not only linked to growth [2, 3]. Indeed, SGA and LGA intercept many neonates with possible intrauterine growth restriction or overgrowth. However, not all the intrauterine restricted growths and overgrowths result in SGA and LGA neonates, and, in contrariwise, some SGA and LGA neonates are constitutionally small or large. The diagnosis of abnormal fetal growth depends on multiple factors, including fetal parameters and accuracy in identifying SGA and LGA neonates. Regarding the latter aspect, the definition of SGA and LGA requires knowledge of gestational age, precise anthropometric measurements at birth, and appropriate reference data for birth weight. Country- or ethnic-specific normative data are important for identifying those at risk. The International Consensus Guideline on SGA [2] recommends the use of national growth charts, when available, or the careful selection of the most appropriate for the region and ethnic-specific population [4], and similar rules should be followed for the identification of LGA.

In 2010, neonatal charts specific for Italian singletons born between 23 and 42 gestational weeks, known as INeS charts, were published [5, 6]. These charts have been widely used for the assessment of neonates and were derived from a nationwide study with a prospective data collection carried out in Italy between 2005 and 2007. The reference set consists of 22,087 girls and 23,375 boys, from 34 centers with a neonatal intensive care unit and neonatology.

The INeS charts were traced separately for first-born and later-born neonates due to a 3% difference in birthweight (BW). To draw these smooth INeS charts, the extended mechanistic growth function (EMGF) method was applied [5]: these charts are completely defined by a function with 10 constants (EGLF-4+1 function), which express the mean pattern of the relation of BW to Gestational Age (GA) according to a prefixed growth model, as well as the conditional standard deviation and skewness of BW. One of the 10 parameters models the difference between first-born and later-born distributions. In a subsequent paper [7], the EMGF approach used to trace the INeS charts was described in detail, and the parameter values of EGLF4 (which differs from EGLF-4+1 only in the absence of the birth-order parameter) were reported.

In some social or emergency contexts, it is not possible to know at the time of delivery the information related to parity. However, on the basis of the above considerations, it is mandatory for the clinical implications to attribute a definition of “high-risk newborn” as reliable as possible.

The aim of this paper, starting from the EGLF-4 function, is to trace birthweight INeS charts for the whole population, i.e., not separated by birth-order. These resulting neonatal charts will serve as a reference when information on parity is unavailable or unreliable, or for better comparisons with other neonatal charts that are not separated by birth-order.

## Methods

### Centiles estimate step

Starting with the EGLF-4 parameter estimates reported by Spada et al. [7], the 3rd, 5th, 10th, 25th, 50th, 75th, 90th, 95th, and 97th centiles of INeS BW charts were computed for the GA range of 23weeks+0days to 42weeks+0days.

### Transformation step

The charts were expressed in terms of smooth GA-specific curves Lambda, Mu and Sigma (L, M, and S), similar to the Cole and Green LMS (GC-LMS) model [8]. The M and S curves correspond to the median (or 50th centile) and coefficient of variation of the BW at each GA, while the L curve accounts for the GA-dependent skewness of the distribution. The values of GA-dependent smooth functions L(t) and S(t) were obtained by fitting the nine centiles estimated in the previous step E(y(t,x,z)), corresponding to a given GA t, sex x, and normal deviate z, with the following function:

1 $$E\left(y\left(t,x,z\right)\right)=M\left(t,x\right)\times\left(1-L\left(t,x\right)\times S\left(t,x\right)\times z\right)^{1/L\left(t,x\right)}$$

Using the function (1), the value of any centile can be computed.

Furthermore, this parametrization allows any BW value (y) of a neonate of a given GA and sex to be expressed as a standard deviation score (SDS):

2 $$SDS=\frac{{\left(\frac{y}{M(t,x)}\right)}^{L(t,x)}-1}{S(t,x)\times L(t,x)}$$

SAS software, version 9.4 (SAS Institute Inc), was used for the analysis.

## Results

**Fig. 1:**
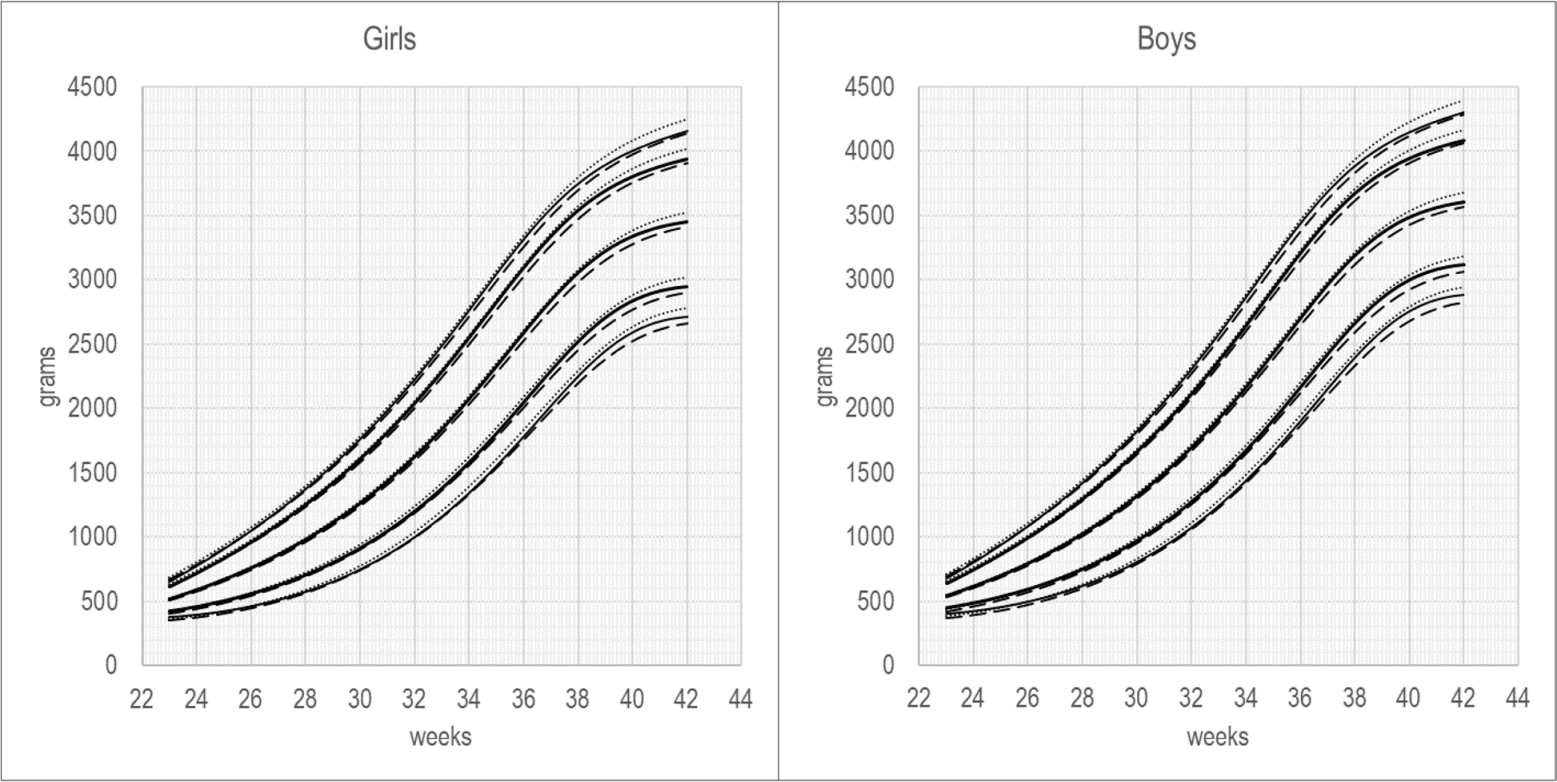
3rd, 10th, 50th, 90th 97th centiles of BW-INeS charts non separated by birth-order (continuous line), compared with BW-INeS charts of first-borns (dashed line) and later-born (dot)

Figure 1 presents the 3^rd^, 10^th^, 50^th^, 90^th^, and 97^th^ centile BW-INeS charts that are not separated by birth order, compared with BW-INeS charts for first- and later-born. As expected, each centile for the non-separated charts falls between the correspondent centile for first-borns and later born. The distances are not equal because the proportion of first-borns changes with GA. All centiles are very close until 32 weeks of gestation, and the max differences of about 100 grams with later born and of about 70 grams with first-borns are observed at term.

**Fig. 2:**
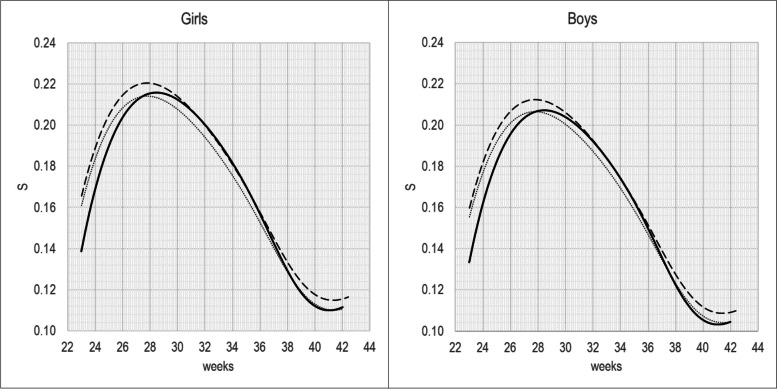
S(t) parameter for BW-INeS charts non-separated by birth-order (continuous line), compared with BW-INeS charts of first-borns (dashed line) and later-born (dot)

**Fig. 3:**
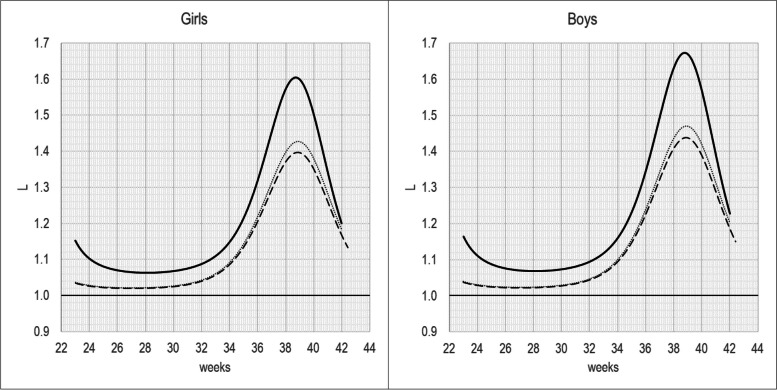
L(t) parameter for BW-INeS charts non-separated by birth-order (continuous line), compared with BW-INeS charts of first-born (dashed line) and later-born (dot)

Figures 2 and 3 show the S and L parameters by GA. The S parameter has a pick at about 29 weeks of gestation for both sexes, while the L is greater than 1 across the entire GA range, indicating a negatively skewed BW distribution, whit a pick at about 39 weeks of gestation for both sexes.

The centiles and the L(t) and S(t) parameters by gestational age were presented in the table A1 (Additional file).

As described in the methods, the values of any centile for each sex at a given GA can be derived from the L, M, S parameters specific to that sex and GA using function (1), and the birth weight of any neonate with known GA and sex can be expressed in SDS using function (2). For example, the value of the 10th centile (whose SDS is -1.28) of BW of a 28 weeks + 0 days girls, we have L=1.0629, M=981, and S=0.2154; therefore,

$$y\left({10}^{th}\right)=981 \times {\left(1 - 1.28\times 1.0629\times 0.2154\right)}^{1/1.0629}=708$$

Alternatively, the BW of 1010 grams of 28 weeks + 0 days girls can be expressed in SDS:

$$SDS=\frac{\left(\frac{1010}{981}\right)^{1.0629}-1}{0.2154\times1.0629}=0.137(\mathrm{corresponding}\;\mathrm{to}\;55\mathrm{th}\;\mathrm{centile}).$$

When gestational age is expressed in whole weeks, the midpoint values of the interval between the indicated week and the following week are recommended (for example: in the case of 28 whole weeks, the values corresponding to 28 weeks + 3 days).

## Discussion

Neonatal INeS charts for birth weight are separated by birth order. The decision to present separate charts for first and later-born neonates was based on a 3% difference between these two populations, which was observed in the initial analysis. This separation allowed for a more accurate assessment of neonates and accounted for the variation in BW between first-born and later-born babies. However, there are scenarios in which the information regarding parity is either missing or unreliable, especially in the multi-ethnic and multicultural society like the current one [8]. In such cases, it becomes essential to have unique charts.

The results of this study show that the centiles for BW-INeS charts that are not separated by birth order fall between the centiles for first-born and later-born neonates, at term, and that the birth weight differences are about 100 grams with later-born neonates and about 70 grams with first-born neonates. Even if such differences are not excessive, the charts presented here can provide a tool with higher accuracy for anthropometric assessment of neonates whose mother's parity is uncertain or unavailable.

In summary, the non-separated BW-INeS charts presented in this paper provide reference values that can be used effectively in clinical practice. The differences between these non-separated charts and those separated by birth order are, for the most part, relatively small, making them a reliable health. Furthermore, the insights into the distribution of birth weights provided by the L and S parameters can aid to standardize BW and to consider it adjusted by sex and gestational age. This is particularly useful both in definition of a neonate as Small or Large for GA as for research analysis.

In addition, an accurate assessment of growth parameters at birth allows better individualization of the management and follow-up of high-risk newborns, with an early identification of diseases related with growth anomalies, such as metabolic, auxological, and neurodevelopmental disorders [9]. These new charts are, then, well integrated with those already existing for some specific categories of newborns, such as twins or other children with genetic disease/malformation syndrome [10–12].

## Conclusion

This study contributes to the ongoing effort to provide accurate and useful tools for neonatal care and demonstrates the flexibility of the INeS charts in accommodating different clinical scenarios. The findings presented here have the potential to enhance the quality of care provided to neonates and support research in the field of neonatology.

### Supplementary Information

**Supplementary Material 1.**

## Data Availability

All data generated or analysed during this study are included in this published article [and its supplementary information files].

## Abbreviations

BW: Birthweight
BW-INeS: BirthWeight-INeS
GC-LMS: Cole and Green LMS model
EMGF: Extendedmechanistic growth function
GA: Gestational Age
INeScharts: Specific neonatal charts for Italian singletons
LGA: Large for gestational age
LMS: Lambda Mu and Sigma
SGA: Small for gestational age
