# Supplementary material for: Italian neonatal birthweight charts derived from INeS not separated by birth order
Source: Ital J Pediatr. 2024 Apr 29;50:89. doi: 10.1186/s13052-024-01660-7 (PMC11057175; doi:10.1186/s13052-024-01660-7)
Supplement: Supplementary file 1 — Supplementary Material 1. [file 13052_2024_1660_MOESM1_ESM.pdf]

Appendix 1. 3rd, 10th, 25th, 50th (M),75th,90th,97th centiles and L(t), S(t) parameter by sex and GA for birthweight. $SDS = \frac{\left(\frac{BW}{M}\right)^{L} - 1}{L \times S}$. Completed weeks has to be compared with bold rows.

GA (wks+days)		Girls									Boys								
wks	days	L	S	3rd	10th	25th	50th (M)	75th	90th	97th	L	S	3rd	10th	25th	50th (M)	75th	90th	97th
23	0	1.1520	0.1386	382	427	471	520	569	611	653	1.1642	0.1334	404	449	494	543	592	635	677
23	1	1.1417	0.1435	384	431	478	530	581	626	670	1.1531	0.1381	406	454	501	553	605	650	695
23	2	1.1328	0.1482	386	436	485	539	593	641	687	1.1435	0.1426	409	459	509	564	617	665	712
**23**	**3**	**1.1250**	**0.1527**	**388**	**440**	**492**	**549**	**606**	**656**	**705**	**1.1352**	**0.1469**	**412**	**464**	**517**	**574**	**630**	**681**	**730**
23	4	1.1182	0.1569	391	445	500	559	618	671	722	1.1279	0.1510	415	470	524	584	643	696	748
23	5	1.1122	0.1610	394	451	507	570	631	686	740	1.1214	0.1549	418	475	532	595	657	712	766
23	6	1.1069	0.1649	397	456	515	580	644	701	757	1.1157	0.1586	422	481	541	606	670	728	784
24	0	1.1022	0.1686	400	461	523	591	657	717	775	1.1107	0.1621	425	487	549	617	684	744	802
24	1	1.0980	0.1721	403	467	531	601	671	733	793	1.1062	0.1655	429	493	558	628	698	760	821
24	2	1.0943	0.1755	407	473	540	612	685	749	812	1.1021	0.1687	433	500	567	640	712	777	840
**24**	**3**	**1.0909**	**0.1786**	**410**	**479**	**548**	**624**	**698**	**765**	**830**	**1.0985**	**0.1718**	**437**	**506**	**576**	**652**	**727**	**794**	**859**
24	4	1.0879	0.1817	414	486	557	635	713	782	849	1.0952	0.1746	442	513	585	663	741	810	878
24	5	1.0852	0.1845	418	492	566	647	727	798	868	1.0923	0.1774	446	520	594	676	756	828	898
24	6	1.0827	0.1872	423	499	575	659	741	815	887	1.0896	0.1799	451	527	604	688	771	845	918
25	0	1.0805	0.1897	427	506	584	671	756	832	907	1.0872	0.1824	456	535	614	701	786	863	938
25	1	1.0784	0.1921	432	513	594	683	771	850	927	1.0850	0.1847	461	543	624	713	802	881	958
25	2	1.0766	0.1944	437	520	604	695	786	867	946	1.0831	0.1868	467	551	634	726	817	899	978
**25**	**3**	**1.0749**	**0.1965**	**442**	**528**	**614**	**708**	**802**	**885**	**967**	**1.0813**	**0.1889**	**473**	**559**	**645**	**740**	**833**	**917**	**999**
25	4	1.0734	0.1985	448	536	624	721	817	903	987	1.0797	0.1908	478	567	656	753	850	936	1020
25	5	1.0721	0.2003	453	544	635	734	833	921	1008	1.0782	0.1925	485	576	667	767	866	955	1041
25	6	1.0708	0.2021	459	552	645	748	849	940	1028	1.0769	0.1942	491	585	678	781	883	974	1063
26	0	1.0697	0.2037	465	561	656	761	865	958	1050	1.0757	0.1957	498	594	690	795	900	993	1084
26	1	1.0687	0.2052	471	569	667	775	882	977	1071	1.0746	0.1971	505	603	702	810	917	1013	1107
26	2	1.0678	0.2065	478	578	679	789	899	997	1093	1.0736	0.1985	512	613	714	825	935	1033	1129
**26**	**3**	**1.0670**	**0.2078**	**485**	**588**	**691**	**804**	**916**	**1016**	**1115**	**1.0727**	**0.1997**	**519**	**623**	**726**	**840**	**952**	**1053**	**1151**
26	4	1.0663	0.2090	492	597	703	819	933	1036	1137	1.0719	0.2008	527	633	739	855	970	1073	1174
26	5	1.0656	0.2100	499	607	715	834	951	1056	1159	1.0713	0.2018	535	643	752	871	989	1094	1197
26	6	1.0651	0.2110	507	617	727	849	969	1077	1182	1.0706	0.2027	543	654	765	887	1007	1115	1221
27	0	1.0646	0.2118	515	627	740	864	987	1097	1205	1.0701	0.2035	552	665	778	903	1026	1136	1244
27	1	1.0642	0.2126	523	638	753	880	1006	1118	1228	1.0697	0.2043	561	676	792	919	1046	1158	1268
27	2	1.0638	0.2133	531	649	767	896	1025	1139	1252	1.0693	0.2049	570	688	806	936	1065	1180	1293
**27**	**3**	**1.0635**	**0.2139**	**540**	**660**	**780**	**913**	**1044**	**1161**	**1276**	**1.0690**	**0.2055**	**579**	**700**	**821**	**953**	**1085**	**1202**	**1317**
27	4	1.0633	0.2144	549	672	794	929	1063	1183	1300	1.0687	0.2059	589	712	835	971	1105	1225	1342
27	5	1.0631	0.2148	558	683	809	946	1083	1205	1324	1.0685	0.2063	599	724	850	989	1125	1248	1368
27	6	1.0630	0.2151	568	695	823	964	1103	1227	1349	1.0684	0.2067	609	737	866	1007	1146	1271	1393
28	0	1.0629	0.2154	578	708	838	981	1123	1250	1374	1.0683	0.2069	620	750	881	1025	1167	1295	1419
28	1	1.0629	0.2156	588	720	853	999	1144	1273	1400	1.0683	0.2071	631	764	897	1044	1189	1318	1446
28	2	1.0629	0.2157	598	733	869	1017	1165	1296	1426	1.0683	0.2072	642	778	914	1063	1211	1343	1472
**28**	**3**	**1.0630**	**0.2157**	**609**	**747**	**885**	**1036**	**1186**	**1320**	**1452**	**1.0684**	**0.2073**	**654**	**792**	**930**	**1082**	**1233**	**1367**	**1499**
28	4	1.0631	0.2157	620	760	901	1055	1208	1344	1478	1.0686	0.2072	666	806	947	1102	1255	1392	1526
28	5	1.0633	0.2156	632	774	917	1074	1230	1369	1505	1.0688	0.2072	678	821	965	1122	1278	1418	1554
28	6	1.0636	0.2155	644	789	934	1094	1252	1393	1532	1.0690	0.2070	691	836	982	1143	1301	1443	1582
29	0	1.0639	0.2153	656	803	951	1114	1275	1419	1560	1.0694	0.2068	704	852	1000	1163	1325	1469	1611
29	1	1.0642	0.2150	668	818	969	1134	1298	1444	1587	1.0697	0.2066	717	868	1019	1185	1349	1496	1639
29	2	1.0646	0.2147	681	834	987	1155	1321	1470	1616	1.0702	0.2063	731	884	1038	1206	1373	1522	1669
**29**	**3**	**1.0651**	**0.2143**	**694**	**850**	**1005**	**1176**	**1345**	**1496**	**1644**	**1.0707**	**0.2059**	**745**	**901**	**1057**	**1228**	**1398**	**1550**	**1698**
29	4	1.0656	0.2139	708	866	1024	1197	1369	1523	1673	1.0712	0.2055	759	918	1076	1251	1423	1577	1728
29	5	1.0662	0.2134	722	882	1043	1219	1394	1550	1703	1.0719	0.2050	774	935	1096	1273	1449	1605	1759
29	6	1.0668	0.2129	736	899	1062	1241	1419	1577	1732	1.0725	0.2045	790	953	1117	1297	1475	1634	1789

GA (wks+days)			Girls										Boys								
wks	days	L	S	3rd	days		L	S	3rd	days		L	S	3rd	days		L	S	3rd	days	97th
30	0		1.0675	0.2123	751	916	1082	1264	1444	1605	1763		1.0733	0.2040	805	971	1138	1320	1501	1662	1820
30	1		1.0683	0.2117	766	934	1102	1287	1470	1633	1793		1.0741	0.2034	821	990	1159	1344	1528	1692	1852
30	2		1.0691	0.2110	782	952	1123	1310	1496	1662	1824		1.0750	0.2027	838	1009	1181	1369	1555	1721	1884
**30**	**3**		**1.0700**	**0.2103**	**798**	**971**	**1144**	**1334**	**1522**	**1691**	**1855**		**1.0760**	**0.2020**	**855**	**1029**	**1203**	**1394**	**1583**	**1751**	**1916**
30	4		1.0710	0.2096	814	990	1165	1358	1549	1720	1887		1.0771	0.2013	872	1049	1225	1419	1611	1782	1949
30	5		1.0721	0.2088	831	1009	1187	1383	1577	1750	1919		1.0783	0.2006	890	1069	1248	1445	1639	1813	1983
30	6		1.0733	0.2079	848	1029	1210	1408	1605	1780	1952		1.0796	0.1998	908	1090	1272	1471	1668	1844	2016
31	0		1.0746	0.2071	866	1049	1232	1434	1633	1811	1985		1.0810	0.1989	927	1111	1295	1498	1697	1876	2050
31	1		1.0760	0.2062	884	1070	1255	1460	1661	1842	2018		1.0825	0.1981	946	1133	1320	1525	1727	1908	2085
31	2		1.0775	0.2052	902	1091	1279	1486	1691	1873	2052		1.0841	0.1972	966	1155	1345	1552	1758	1941	2120
**31**	**3**		**1.0791**	**0.2043**	**921**	**1112**	**1303**	**1513**	**1720**	**1905**	**2086**		**1.0859**	**0.1962**	**986**	**1178**	**1370**	**1580**	**1788**	**1974**	**2155**
31	4		1.0809	0.2033	940	1134	1328	1540	1750	1937	2121		1.0878	0.1953	1007	1201	1396	1609	1819	2007	2191
31	5		1.0828	0.2022	960	1157	1353	1568	1780	1970	2156		1.0898	0.1943	1028	1225	1422	1638	1851	2041	2227
31	6		1.0848	0.2012	981	1180	1378	1596	1811	2003	2191		1.0921	0.1932	1049	1249	1448	1667	1883	2076	2264
32	0		1.0871	0.2000	1001	1203	1404	1625	1843	2037	2227		1.0945	0.1922	1071	1274	1476	1697	1916	2111	2301
32	1		1.0895	0.1989	1023	1227	1430	1654	1874	2071	2263		1.0971	0.1911	1094	1299	1503	1728	1949	2146	2339
32	2		1.0921	0.1977	1045	1251	1457	1683	1907	2105	2300		1.1000	0.1900	1117	1325	1532	1758	1982	2182	2377
**32**	**3**		**1.0950**	**0.1966**	**1067**	**1276**	**1485**	**1713**	**1939**	**2140**	**2337**		**1.1031**	**0.1888**	**1140**	**1351**	**1560**	**1790**	**2016**	**2218**	**2415**
32	4		1.0980	0.1953	1090	1301	1513	1744	1972	2176	2374		1.1064	0.1876	1165	1377	1589	1822	2051	2255	2454
32	5		1.1014	0.1941	1113	1327	1541	1775	2006	2211	2412		1.1101	0.1864	1189	1405	1619	1854	2086	2292	2493
32	6		1.1050	0.1928	1137	1354	1570	1806	2040	2247	2450		1.1140	0.1852	1214	1432	1649	1887	2121	2329	2532
33	0		1.1089	0.1915	1161	1380	1599	1838	2074	2284	2489		1.1183	0.1839	1240	1461	1680	1920	2157	2367	2572
33	1		1.1132	0.1901	1186	1408	1629	1870	2109	2320	2527		1.1229	0.1826	1266	1489	1711	1954	2193	2405	2612
33	2		1.1178	0.1887	1211	1436	1659	1903	2144	2358	2566		1.1280	0.1813	1293	1519	1743	1988	2229	2444	2653
**33**	**3**		**1.1228**	**0.1873**	**1237**	**1464**	**1690**	**1936**	**2179**	**2395**	**2606**		**1.1334**	**0.1799**	**1320**	**1548**	**1775**	**2023**	**2266**	**2483**	**2694**
33	4		1.1283	0.1859	1263	1493	1721	1970	2215	2433	2645		1.1393	0.1785	1348	1579	1808	2058	2304	2522	2735
33	5		1.1342	0.1844	1290	1522	1753	2004	2251	2471	2685		1.1457	0.1771	1376	1610	1841	2093	2342	2561	2776
33	6		1.1406	0.1829	1317	1552	1785	2039	2288	2509	2725		1.1527	0.1757	1405	1641	1875	2129	2380	2601	2817
34	0		1.1475	0.1814	1345	1582	1817	2073	2325	2548	2765		1.1602	0.1742	1434	1673	1909	2166	2418	2641	2859
34	1		1.1549	0.1799	1373	1613	1850	2109	2362	2587	2805		1.1683	0.1727	1464	1705	1943	2203	2457	2682	2901
34	2		1.1630	0.1783	1402	1644	1884	2144	2400	2626	2846		1.1771	0.1711	1495	1738	1979	2240	2496	2722	2942
**34**	**3**		**1.1717**	**0.1766**	**1431**	**1676**	**1918**	**2180**	**2437**	**2665**	**2886**		**1.1866**	**0.1695**	**1526**	**1771**	**2014**	**2277**	**2535**	**2763**	**2984**
34	4		1.1810	0.1750	1461	1708	1952	2216	2475	2704	2926		1.1968	0.1679	1557	1805	2050	2315	2574	2804	3026
34	5		1.1911	0.1733	1491	1741	1986	2253	2513	2743	2967		1.2078	0.1663	1589	1839	2086	2353	2614	2844	3068
34	6		1.2019	0.1715	1522	1774	2021	2289	2551	2782	3007		1.2196	0.1646	1621	1874	2123	2391	2654	2885	3110
35	0		1.2134	0.1698	1553	1807	2056	2326	2590	2822	3047		1.2323	0.1629	1654	1909	2159	2430	2694	2926	3151
35	1		1.2258	0.1680	1585	1841	2092	2363	2628	2861	3087		1.2458	0.1611	1687	1944	2197	2469	2734	2967	3192
35	2		1.2390	0.1662	1617	1875	2128	2400	2666	2900	3126		1.2603	0.1593	1721	1980	2234	2507	2773	3007	3233
**35**	**3**		**1.2530**	**0.1643**	**1649**	**1909**	**2163**	**2438**	**2704**	**2939**	**3165**		**1.2756**	**0.1575**	**1755**	**2016**	**2272**	**2546**	**2813**	**3047**	**3274**
35	4		1.2678	0.1624	1682	1944	2200	2475	2742	2977	3204		1.2919	0.1557	1790	2053	2309	2585	2853	3087	3314
35	5		1.2834	0.1605	1715	1978	2236	2512	2780	3015	3242		1.3091	0.1538	1825	2089	2347	2624	2892	3127	3354
35	6		1.2998	0.1586	1748	2013	2272	2549	2818	3053	3280		1.3272	0.1519	1860	2126	2385	2663	2931	3166	3393
36	0		1.3170	0.1566	1782	2049	2308	2586	2855	3090	3317		1.3462	0.1499	1895	2163	2423	2702	2970	3205	3431
36	1		1.3349	0.1546	1815	2084	2345	2623	2892	3127	3354		1.3659	0.1480	1931	2200	2461	2740	3009	3243	3469
36	2		1.3534	0.1526	1849	2119	2381	2660	2929	3164	3390		1.3864	0.1460	1966	2237	2499	2778	3047	3281	3506
**36**	**3**		**1.3725**	**0.1506**	**1883**	**2154**	**2417**	**2696**	**2965**	**3199**	**3425**		**1.4076**	**0.1440**	**2002**	**2274**	**2537**	**2816**	**3085**	**3318**	**3542**
36	4		1.3920	0.1486	1918	2190	2452	2732	3000	3234	3459		1.4293	0.1420	2038	2311	2574	2854	3122	3355	3578
36	5		1.4118	0.1465	1952	2225	2488	2767	3035	3269	3492		1.4513	0.1400	2074	2348	2611	2890	3158	3390	3613
36	6		1.4317	0.1445	1986	2259	2523	2802	3070	3302	3525		1.4736	0.1380	2110	2385	2648	2927	3194	3425	3646

GA (wks+days)		Girls									Boys								
wks	days	L	S	3rd	10th	25th	50th (M)	75th	90th	97th	L	S	3rd	10th	25th	50th (M)	75th	90th	97th
37	0	1.4516	0.1425	2020	2294	2557	2836	3103	3335	3556	1.4959	0.1360	2146	2421	2684	2963	3228	3459	3679
37	1	1.4713	0.1405	2054	2328	2591	2870	3136	3367	3587	1.5181	0.1340	2182	2457	2720	2998	3263	3492	3711
37	2	1.4905	0.1385	2088	2362	2625	2902	3168	3397	3617	1.5397	0.1320	2217	2492	2755	3032	3296	3524	3741
**37**	**3**	**1.5090**	**0.1365**	**2121**	**2395**	**2658**	**2934**	**3199**	**3427**	**3645**	**1.5607**	**0.1300**	**2253**	**2527**	**2789**	**3065**	**3328**	**3555**	**3771**
37	4	1.5266	0.1345	2154	2428	2690	2966	3229	3456	3673	1.5808	0.1281	2287	2562	2823	3098	3359	3585	3800
37	5	1.5429	0.1326	2186	2460	2721	2996	3258	3484	3700	1.5996	0.1262	2322	2595	2856	3130	3390	3614	3827
37	6	1.5579	0.1308	2218	2491	2751	3025	3286	3511	3726	1.6168	0.1244	2355	2628	2888	3160	3419	3642	3854
38	0	1.5711	0.1290	2250	2522	2781	3054	3313	3537	3750	1.6323	0.1226	2389	2660	2919	3190	3447	3669	3879
38	1	1.5823	0.1273	2281	2551	2809	3081	3339	3562	3774	1.6456	0.1208	2421	2691	2949	3218	3474	3694	3904
38	2	1.5915	0.1256	2311	2580	2837	3107	3364	3586	3797	1.6566	0.1192	2453	2722	2977	3246	3500	3719	3927
**38**	**3**	**1.5982**	**0.1240**	**2340**	**2608**	**2863**	**3132**	**3388**	**3609**	**3819**	**1.6650**	**0.1176**	**2483**	**2751**	**3005**	**3272**	**3525**	**3743**	**3950**
38	4	1.6024	0.1224	2368	2634	2889	3156	3411	3630	3840	1.6706	0.1160	2513	2779	3032	3297	3549	3765	3971
38	5	1.6041	0.1210	2395	2660	2913	3179	3433	3651	3860	1.6734	0.1146	2542	2806	3057	3321	3571	3787	3992
38	6	1.6030	0.1196	2422	2685	2936	3201	3453	3671	3879	1.6731	0.1132	2570	2832	3081	3344	3593	3807	4012
39	0	1.5991	0.1183	2447	2708	2958	3222	3473	3690	3897	1.6697	0.1119	2597	2856	3104	3365	3613	3827	4031
39	1	1.5926	0.1171	2471	2730	2979	3241	3492	3708	3915	1.6633	0.1107	2622	2880	3126	3386	3633	3846	4049
39	2	1.5834	0.1160	2494	2751	2998	3260	3509	3725	3932	1.6539	0.1096	2646	2902	3147	3405	3651	3864	4066
**39**	**3**	**1.5717**	**0.1150**	**2516**	**2771**	**3017**	**3277**	**3526**	**3742**	**3948**	**1.6417**	**0.1086**	**2670**	**2923**	**3166**	**3423**	**3668**	**3880**	**4083**
39	4	1.5577	0.1141	2536	2790	3034	3294	3542	3757	3964	1.6268	0.1076	2691	2943	3185	3441	3685	3897	4099
39	5	1.5416	0.1133	2556	2807	3051	3309	3557	3772	3979	1.6094	0.1068	2712	2961	3202	3457	3700	3912	4114
39	6	1.5235	0.1126	2574	2824	3066	3323	3571	3786	3993	1.5898	0.1061	2731	2979	3218	3472	3715	3926	4128
40	0	1.5038	0.1119	2591	2839	3080	3337	3584	3800	4007	1.5683	0.1054	2749	2995	3233	3486	3729	3940	4143
40	1	1.4828	0.1114	2606	2853	3093	3350	3597	3813	4020	1.5452	0.1048	2766	3010	3247	3499	3742	3953	4156
40	2	1.4607	0.1109	2621	2866	3105	3361	3609	3825	4033	1.5207	0.1044	2781	3024	3259	3511	3754	3966	4169
**40**	**3**	**1.4379**	**0.1106**	**2634**	**2878**	**3116**	**3372**	**3620**	**3836**	**4045**	**1.4954**	**0.1040**	**2795**	**3036**	**3271**	**3523**	**3766**	**3978**	**4182**
40	4	1.4145	0.1103	2646	2889	3127	3382	3630	3847	4057	1.4694	0.1037	2808	3048	3282	3533	3776	3989	4194
40	5	1.3909	0.1101	2657	2899	3136	3392	3640	3858	4069	1.4430	0.1034	2820	3058	3292	3543	3787	4000	4206
40	6	1.3673	0.1100	2667	2908	3145	3401	3649	3868	4080	1.4166	0.1033	2830	3068	3301	3552	3796	4010	4217
41	0	1.3439	0.1099	2675	2916	3153	3409	3658	3878	4091	1.3904	0.1032	2840	3077	3309	3561	3805	4020	4228
41	1	1.3210	0.1099	2683	2923	3160	3416	3666	3887	4101	1.3646	0.1032	2848	3084	3317	3568	3814	4030	4239
41	2	1.2987	0.1100	2690	2929	3166	3423	3674	3896	4111	1.3394	0.1032	2855	3091	3324	3576	3822	4039	4249
**41**	**3**	**1.2771**	**0.1102**	**2696**	**2935**	**3172**	**3429**	**3682**	**3905**	**4121**	**1.3151**	**0.1034**	**2862**	**3097**	**3330**	**3582**	**3829**	**4048**	**4259**
41	4	1.2564	0.1104	2700	2940	3177	3435	3689	3913	4131	1.2916	0.1036	2867	3102	3335	3588	3837	4056	4269
41	5	1.2366	0.1107	2704	2944	3181	3440	3695	3921	4141	1.2692	0.1038	2872	3107	3340	3594	3843	4064	4279
41	6	1.2178	0.1110	2708	2947	3185	3445	3701	3929	4150	1.2479	0.1041	2875	3110	3344	3599	3850	4072	4288
42	0	1.2000	0.1114	2710	2950	3189	3450	3707	3936	4159	1.2278	0.1045	2878	3113	3348	3604	3856	4079	4298
